# Difference in the distribution of tumor‐infiltrating CD8+ T cells and FOXP3+ T cells between micronodular thymoma with lymphoid stroma and micronodular thymic carcinoma with lymphoid stroma

**DOI:** 10.1111/pin.13099

**Published:** 2021-04-05

**Authors:** Haruna Yagi, Masato Nakaguro, Masafumi Ito, Yuki Okumura, Seishiro Takahashi, Yoichiro Aoshima, Yasunori Enomoto, Shiori Meguro, Hideya Kawasaki, Isao Kosugi, Yoshie Shimoyama, Hiroshi Ogawa, Hisashi Tateyama, Toshihide Iwashita

**Affiliations:** ^1^ Department of Regenerative and Infectious Pathology Hamamatsu University School of Medicine Hamamatsu Japan; ^2^ Department of Pathology and Laboratory Medicine Nagoya University Hospital Nagoya Japan; ^3^ Department of Pathology, Japanese Red Cross Nagoya First Hospital Nagoya Japan; ^4^ Department of Diagnostic Pathology Seirei Mikatahara Hospital Hamamatsu Japan; ^5^ Institute for NanoSuit Research, Preeminent Medical Photonics Education and Research Center Hamamatsu University School of Medicine Hamamatsu Japan; ^6^ Department of Pathology, Clinical Laboratory Kasugai Municipal Hospital Kasugai Japan

**Keywords:** CD8, FOXP3, micronodular thymic carcinoma with lymphoid stroma, micronodular thymoma with lymphoid stroma, PD‐1, PD‐L1, thymic epithelial tumor, tumor microenvironment, tumor‐infiltrating lymphocyte

## Abstract

Micronodular thymoma with lymphoid stroma (MNT) is a rare thymic epithelial neoplasm subtype characterized by a micronodular tumor cell growth pattern and abundant lymphoid stroma. Micronodular thymic carcinoma with lymphoid stroma (MNCA) is considered as a malignant counterpart of MNT and exhibits a growth pattern similar to that of MNT but has histologic features reminiscent of thymic squamous cell carcinoma, such as cytologic atypia and CD5 and CD117 immunoexpression. Although both MNT and MNCA are characterized by abundant lymphoid stroma, it remains unknown whether there are differences in infiltrating lymphocytes between MNT and MNCA. We analyzed the immune microenvironment profile in eight MNT and three MNCA cases. The cell density of CD8‐positive T cells was significantly higher in MNT than in MNCA, whereas that of FOXP3‐positive T cells was significantly higher in MNCA than in MNT. There was no significant difference in the cell density of programmed death protein 1‐positive T cells and programmed death ligand 1 expression between the MNT and MNCA cases. Our findings indicated that the immune microenvironment of MNCA differed from that of MNT and, compared with the T‐cell profile of MNT, that of MNCA was more suppressive to patients′ antitumor immune response.

**Abbreviations**
HPF: high‐power field
ICI: immune checkpoint inhibitor
MNCA: micronodular thymic carcinoma with lymphoid stroma
MNT: micronodular thymoma with lymphoid stroma
PD‐1: programmed death protein 1
PD‐L1: programmed death ligand 1
SCC: squamous cell carcinoma
TIL: tumor‐infiltrating lymphocyte

## INTRODUCTION

Thymic epithelial neoplasms are classified into thymoma (types A, AB, B1, B2 and B3), thymic squamous cell carcinoma (SCC), and other rare subtypes in the *World Health Organization (WHO) Classification of Tumors of the Lung, Pleura, Thymus and Heart 4th Edition*.^1^ Micronodular thymoma with lymphoid stroma (MNT) is a rare subtype that accounts for 1–5% of all thymic epithelial neoplasms characterized by a micronodular tumor cell growth pattern and abundant lymphoid stroma with germinal centers.^2^, ^3^, ^4^, ^5^, ^6^ Micronodular thymic carcinoma with lymphoid stroma (MNCA) is an extremely rare subtype of thymic epithelial neoplasm, and only 20 cases have been reported so far in previous English publications.^4^ MNCA is also characterized by micronodular cell proliferation and abundant lymphoid stroma, similar to MNT. However, in contrast to MNT, MNCA has thymic SCC‐like features, such as cytologic atypia, increased mitotic activity, and CD5 and CD117 (c‐kit) expression.^2^, ^4^, ^5^, ^6^ In one study, 25% of MNCA cases had an adjacent MNT that transitioned from the MNCA.^4^ Although these findings suggest that MNCA is a malignant counterpart of MNT,^4^, ^5^ the clinical features of MNCA and the relationship between MNT and MNCA are largely unknown due to the extreme rarity of MNCA.

Lymphoid stroma is generally associated with host immune response to tumor antigens.^7^, ^8^ The lymphoid stroma of MNT has a larger number of Langerhans cells and mature dendritic cells than type A and AB thymomas, suggesting the presence of a specific tumor immune microenvironment in MNT.^7^ In recent years, a large number of studies have investigated the tumor immune microenvironment, including tumor‐infiltrating lymphocytes (TILs), such as CD8 (+) cytotoxic T cells, FOXP3 (+) regulatory T cells, programmed death protein 1 (PD‐1) (+) T cells, and the programmed death ligand 1 (PD‐L1) expression in tumor cells.^9^, ^10^, ^11^ While previous studies have explored TILs and PD‐L1 expression in typical thymoma and thymic SCC cases, no studies have focused on TILs and PD‐L1 expression in MNT and MNCA.

This study aimed to investigate the immune microenvironment, including the distribution and cell density of CD8 (+) T cells, FOXP3 (+) T cells and PD‐1 (+) T cells and PD‐L1 expression in tumor cells from patients with MNT and MNCA.

## MATERIALS AND METHODS

### Patients and tissue specimens

We retrieved eight MNT and three MNCA cases, all of which were surgically resected at Seirei Mikatahara Hospital, Nagoya University Hospital and Japanese Red Cross Nagoya First Hospital in Japan between 2006 and 2019. The histologic diagnosis was reconfirmed by two board‐certified pathologists (HY and TI) based on the WHO criteria.^2^ To distinguish between MNT and MNCA, we examined the presence of nuclear atypia, the mitotic counts of tumor cells, and CD5 and CD117 expression using immunohistochemistry. For the comparative analysis, data and tissue specimens of 21 patients with common types of thymoma (four with type A, six with type AB, five with type B1, four with type B2 and two with type B3) and three patients with thymic SCC were also retrieved. The extracted patient data included patient age, sex, surgical procedure, Masaoka stage,^1^ TNM stage based on the *TNM Classification of Malignant Tumours 8th Edition of the UICC*,^12^ and clinical outcomes. The clinical courses of two cases (Cases 2 and 11) were previously published as case reports.^13^, ^14^

This study was conducted in accordance with the recommendations of the Declaration of Helsinki and was approved by the ethics committees of the participating hospitals.

### Histological and immunohistochemical staining

CD8, FOXP3, PD‐1, CD117 and CD5 immunohistochemistry was performed in the eight MNT and three MNCA cases. PD‐L1 immunohistochemistry was performed in all the 35 thymic epithelial neoplasm cases. Antigen retrieval for CD8, FOXP3 and PD‐L1 was performed with the buffer preheated to 95°C for 40 min (pH 9.0). Antigen retrieval for PD‐1, CD117 and CD5 was conducted with preheating to 120°C for 15 min (pH 6.0). The antibodies against these antigens were as follows: CD8 (clone 4B11, 1:40; Leica Biosystems, Wetzlar, Germany), FOXP3 (clone 236 A/E7, 1:100; Abcam, Cambridge, UK), PD‐1 (clone NAT105, 1:50; Abcam), PD‐L1 (clone E1L3N, 1:100; Cell Signaling Technology, Danvers, MA, USA), CD117 (polyclonal, 1:500; DakoCytomation, Carpinteria, CA, USA) and CD5 (clone 4C7, 1:400; Leica Biosystems). All sections were incubated with primary antibodies for 30 min. They were then incubated with peroxidase‐labeled goat anti‐mouse/rabbit IgG antibody (Nichirei Biosciences, Tokyo, Japan) for 30 min. The immune response was visualized using 3,3′‐diaminobenzidine (DakoCytomation) and counterstained with hematoxylin. Two board‐certified pathologists (HY and TI) examined the hematoxylin and eosin and immunohistochemical stains for CD5 and CD117.

### Scoring

To clarify the localization of the infiltrating CD8 (+) T cells, FOXP3 (+) T cells and PD‐1 (+) T cells, we performed chromogenic double staining for MNT and MNCA using anti‐CD8, FOXP3 or PD‐1 antibody and peroxidase‐conjugated Universal Immuno‐enzyme polymer anti‐mouse solution, stained with 3,3′‐diaminobenzidine + substrate‐chromogen, followed by treatment with anti‐AE1/AE3 antibody and alkaline phosphatase‐conjugated Universal Immuno‐enzyme polymer anti‐mouse solution (Nichirei Biosciences), which was stained bluish‐purple with fast blue BB salt (Sigma‐Aldrich, St. Louis, MO, USA). A tumor nest was defined as an AE1/AE3‐positive area, while a peri‐tumoral lymphoid stroma was defined as an AE1/AE3‐negative peri‐tumoral lymphocyte‐rich area (Supplementary Fig. S1).

Ten high‐power fields (HPF, ×400) of the tumor nest and peri‐tumoral lymphoid stroma were randomly selected for MNT and MNCA, and photographs including both tumor nest and peri‐tumoral lymphoid stroma were taken. The cell densities of the infiltrating CD8 (+) T cells, FOXP3 (+) T cells and PD‐1 (+) T cells (per mm^2^) in the tumor nest and peri‐tumoral lymphoid stroma in each photograph were measured and the average was calculated (Supplementary Fig. S1). Lymphoid follicles were excluded from the photograph to evaluate the stroma in the identical histomorphologic settings. The TIL densities in the tumor nest and peri‐tumoral lymphoid stroma were analyzed separately to avoid bias due to the difference in densities of TILs in these areas. The CD8/FOXP3 ratio of the tumor nest and peri‐tumoral lymphoid stroma in each case was defined as the density of CD8 (+) T cells divided by the density of FOXP3 (+) T cells.

The PD‐L1 positivity rate was defined as the percentage resulting from dividing the number of PD‐L1 (+) tumor cells by the number of all tumor cells in each photograph. Two pathologists independently calculated and averaged the PD‐L1 positivity rate in three different HPFs for each tumor.

### Statistical analysis

Student's t‐tests were used to compare the cell densities of TILs (CD8 (+) T cells, FOXP3 (+) T cells and PD‐1 (+) T cells) and the CD8/FOXP3 ratio between the MNT and MNCA cases and also to compare the PD‐L1 positivity rates across the MNT, MNCA, typical thymoma and thymic SCC cases. *P*‐values of < 0.05 were considered statistically significant.

## RESULTS

### Clinical features

The clinical findings are summarized in Table 1. The mean ages of the MNT and MNCA patients were 63 years (range, 47–76 years) and 64 years (range, 45–76 years), respectively. The MNT group comprised four men and four women, and the MNCA group two men and one woman. No cases of myasthenia gravis were observed. The tumor size ranged from 18 to 60 mm (mean, 39 mm) in the MNT group and 16 to 20 mm (mean, 18 mm) in the MNCA group. According to the Masaoka staging system,^1^ two patients with MNT and two with MNCA were classified as having Stage I disease, and six patients with MNT and one with MNCA were classified as having Stage II disease. According to TNM classification^12^, all patients with MNT and MNCA were classified as having Stage I disease. In the follow‐up period (13–120 months, median 58.7 months), one MNT patient showed local recurrence about 10 years after partial resection.

**Table 1:** Clinicopathological characteristics of eight MNT and three MNCA cases

Case	Age (years)	Sex	Tumor size (mm)	Diagnosis	Operation	Masaoka stage	TNM classification	Follow‐up (months)	Outcome
1	62	M	40	MNT	Thymothymectomy	I	I	75	No recurrence
2	59	F	34	MNT	Thymomectomy	II	I	120	Recurrence at 10 years after operation
3	74	M	35	MNT	Thymomectomy	II	I	24	No recurrence
4	76	F	30	MNT	Thymothymectomy	II	I	72	No recurrence
5	58	M	18	MNT	Thymothymectomy	II	I	74	No recurrence
6	76	F	60	MNT	Thymothymectomy	II	I	14	No recurrence
7	47	F	55	MNT	Thymothymectomy	II	I	84	No recurrence
8	54	M	40	MNT	Thymomectomy	I	I	30	No recurrence
9	76	M	16	MNCA	Thymomectomy	II	I	48	No recurrence
10	71	M	20	MNCA	Thymomectomy	I	I	13	No recurrence
11	45	F	18	MNCA	Thymomectomy	I	I	92	No recurrence

MNCA, micronodular thymic carcinoma with lymphoid stroma; MNT, micronodular thymoma with lymphoid stroma.

### Histological findings

**Figure 1:**
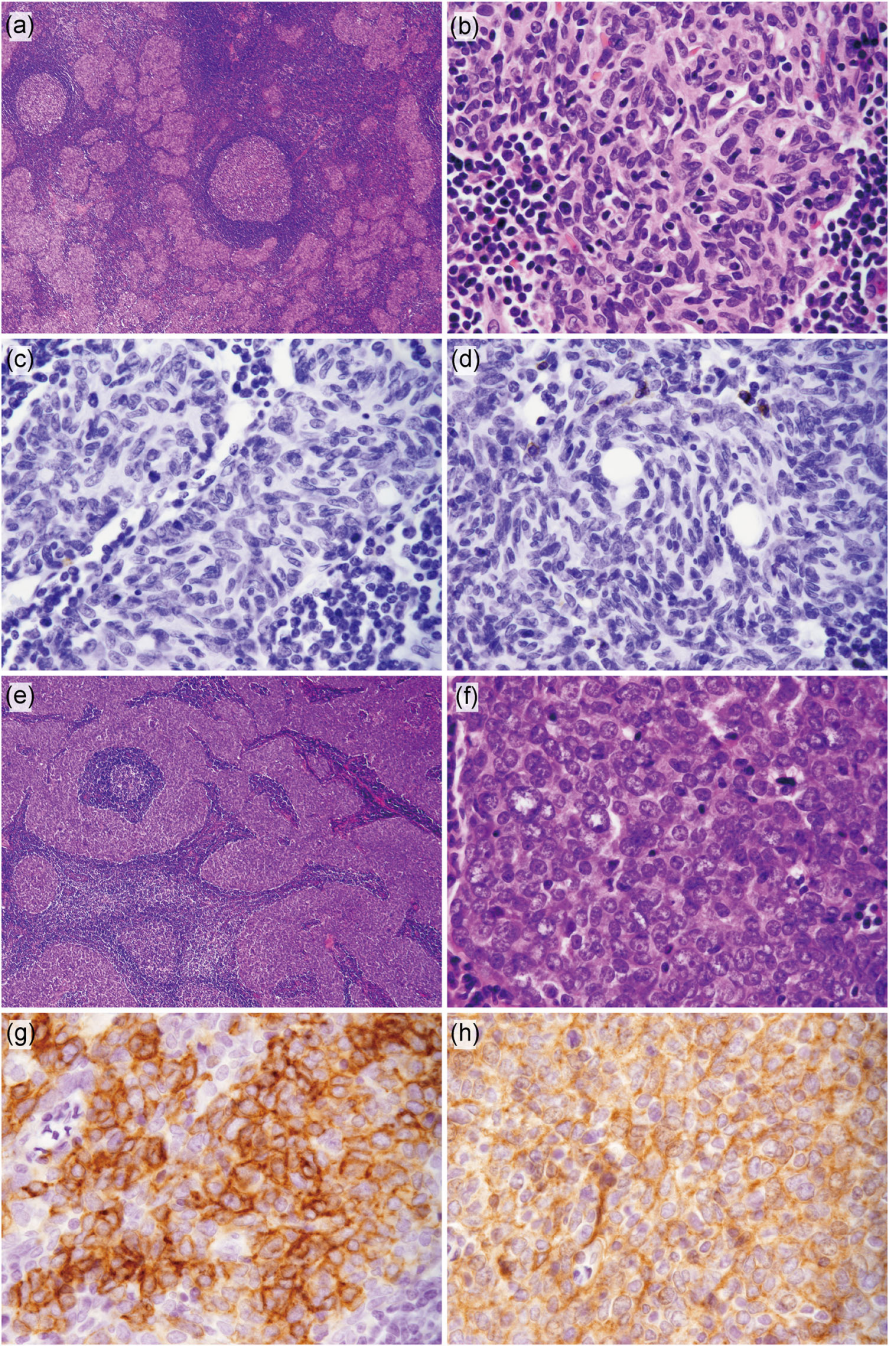
Microscopic appearance of micronodular thymoma with lymphoid stroma (MNT) and micronodular thymic carcinoma with lymphoid stroma (MNCA). (**a,e**) Representative examples of low‐magnification images of MNT and MNCA on hematoxylin and eosin staining. (**a**) MNT and (**e**) MNCA showing a micronodular pattern separated by abundant lymphoid stroma with germinal centers. (**b,f**) Representative examples of high‐magnification images of MNT and MNCA on hematoxylin and eosin staining. (**b**) Tumor nests of MNT comprising spindle‐ or oval‐shaped epithelial tumor cells with uniform nuclei containing indistinct nucleoli. (**f**) Tumor cells of MNCA were rounder and larger than those of MNT and had vesicular nuclei containing distinct nucleoli. (**c,g**) Representative examples of high‐magnification images of immunohistochemical staining for CD117. (**c**) In MNT, tumor cells were negative for CD117. (**g**) In MNCA, tumor cells were positive for CD117. (**d,h**) Representative examples of high‐magnification images of immunohistochemical staining for CD5. (**d**) In MNT, tumor cells were negative for CD5, while infiltrating T cells were positive for CD5. (**h**) In MNCA, tumor cells were positive for CD5.

The representative histological and immunohistochemical features are shown in Fig. 1. Both MNT and MNCA showed micronodular tumor cell proliferation separated by abundant lymphoid stroma with germinal centers (Fig. 1a,e). The tumor nests in MNT comprised spindle‐ or oval‐shaped epithelial tumor cells with uniform nuclei containing indistinct nucleoli (Fig. 1b). The mitotic counts ranged from 0 to 2 per HPF. Immunohistochemically, the tumor cells were negative for CD117 (Fig. 1c) and CD5 (Fig. 1d). On the other hand, the tumor cells of MNCA had round, vesicular, and larger nuclei than those of MNT (Fig. 1f) and had mitotic counts ranging from 3 to 6 per HPF. The tumor cells were diffusely positive for CD117 (Fig. 1g) in all MNCA cases. Two cases were diffusely positive for CD5, whereas one case showed partial positivity (Fig. 1h).

### Distribution and cell density of infiltrating CD8 (+), FOXP3 (+) and PD‐1 (+) T cells

**Figure 2:**
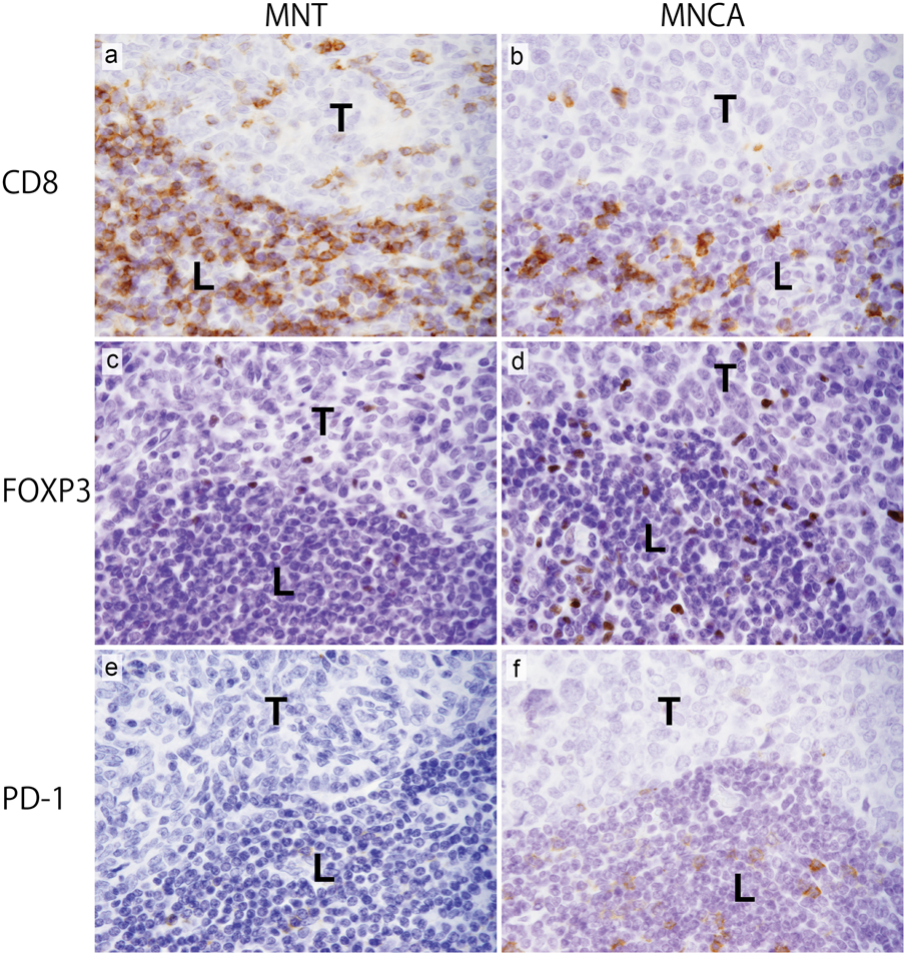
CD8 (+) T cells, FOXP3 (+) T cells and PD‐1 (+) T cell infiltration in micronodular thymoma with lymphoid stroma (MNT) and micronodular thymic carcinoma with lymphoid stroma (MNCA). (**a,b**) Representative examples of immunohistochemical staining for CD8. (**a**) MNT. (**b**) MNCA. (**c,d**) Representative examples of immunohistochemical staining for FOXP3. (**c)** MNT. (d) MNCA. (**e,f)** Representative examples of immunohistochemical staining for programmed death protein 1 (PD‐1). (**e**) MNT. (**f**) MNCA. T, tumor nest; L, peri‐tumoral lymphoid stroma.

**Figure 3:**
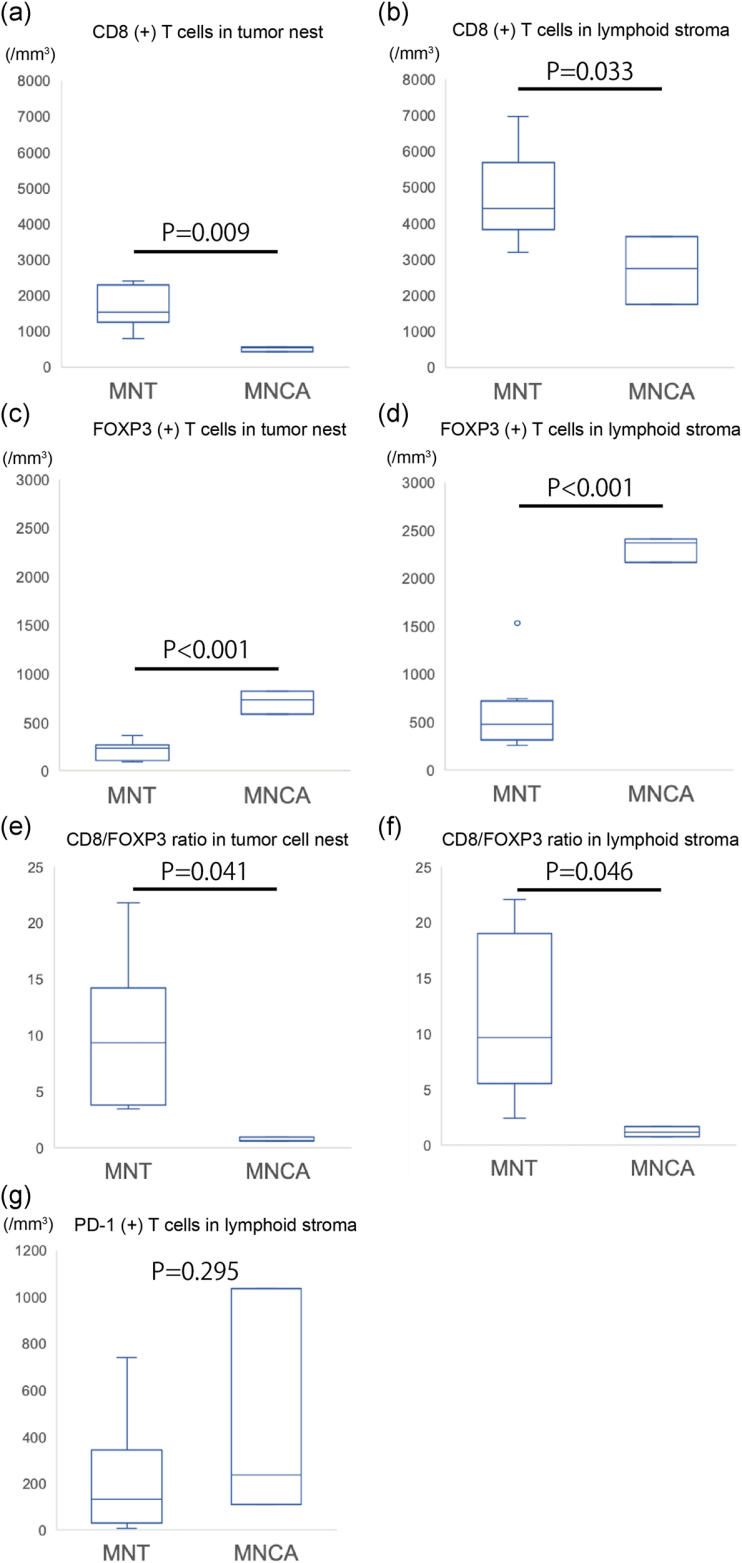
Distribution and cell density of infiltrating CD8 (+) T cells, FOXP3 (+) T cells and programmed death protein 1 (PD‐1) (+) T cells and CD8/FOXP3 ratio in micronodular thymoma with lymphoid stroma (MNT) and micronodular thymic carcinoma with lymphoid stroma (MNCA). (**a,b**) Cell density of CD8 (+) T cells (per mm^2^) in the tumor nest and peri‐tumoral lymphoid stroma of MNT (*n* = 8) and MNCA (*n* = 3). (**c,d**) Cell density of FOXP3 (+) T cells (per mm^2^) in the tumor nest and peri‐tumoral lymphoid stroma of MNT (*n* = 8) and MNCA (*n* = 3). (**e,f**) CD8/FOXP3 ratio in the tumor nest and peri‐tumoral lymphoid stroma of MNT (*n* = 8) and MNCA (*n* = 3). (**g**) Cell density of PD‐1 (+) T cells (per mm^2^) in the peri‐tumoral lymphoid stroma of MNT (*n* = 8) and MNCA (*n* = 3). (**a–g**) Boxes represent the interquartile range, with the upper whisker indicating the 75th percentile and the lower whisker indicating the 25th percentile. The median values are indicated by a horizontal line.

Representative immunohistochemical features are shown in Fig. 2, and the results are summarized in Table 2 and Fig. 3.

**Table 2:** Cell density of CD8 (+) T cells, FOXP3 (+) T cells and PD‐1 (+) T cells and CD8/FOXP3 ratio

	MNT	MNCA	*P*‐value
	(*n* = 8)	(*n* = 3)	
Density of CD8 (+) immune cells (/mm^2^)			
Tumor nest	1660 ± 545	513 ± 54	**0.009**
Peri‐tumoral lymphoid stroma	4746 ± 1171	2712 ± 773	**0.033**
Density of FOXP3 (+) immune cells (/mm^2^)			
Tumor nest	214 ± 89	715 ± 95	**<0.001**
Peri‐tumoral lymphoid stroma	599 ± 390	2318 ± 106	**<0.001**
CD8/FOXP3 ratio			
Tumor nest	9.8 ± 6.0	0.7 ± 0.2	**0.041**
Peri‐tumoral lymphoid stroma	11.4 ± 6.9	1.2 ± 0.4	**0.046**
Density of PD‐1 (+) immune cells (/mm^2^)			
Peri‐tumoral lymphoid stroma	219 ± 232	461 ± 411	0.295

MNCA, micronodular thymic carcinoma with lymphoid stroma; MNT, micronodular thymoma with lymphoid stroma; PD‐1, programmed death protein 1.

In the normal thymic tissue, CD8 (+) T cells were observed in both the cortex and medulla. Conversely, FOXP3 (+) T cells and PD‐1 (+) cells were mainly observed in the medulla, with small amounts in the cortex (Supplementary Fig. S2). These findings are consistent with those of previously published studies.^15^, ^16^

We compared the cell densities of CD8 (+) T cells and FOXP3 (+) T cells in the tumor nest and peri‐tumoral lymphoid stroma between MNT and MNCA cases. The cell density of CD8 (+) T cells was higher in MNT than in MNCA in both the tumor nest (*P* = 0.009) and peri‐tumoral lymphoid stroma (*P* = 0.033) (Figs. 2, 3; Table 2). FOXP3 (+) T cells in the tumor nest and peri‐tumoral lymphoid stroma was significantly higher in MNCA than MNT (tumor nest, *P* < 0.001; peri‐tumoral lymphoid stroma, *P* < 0.001) (Figs. 2, 3; Table 2). As a result, the CD8/FOXP3 ratio was significantly higher in MNT cases than in MNCA cases in both the tumor nest (*P* = 0.041) and peri‐tumoral lymphoid stroma (*P* = 0.046) (Fig. 3e,f; Table 2).

Next, we compared the distribution and cell density of PD‐1 (+) T cells in the tumor nest and peri‐tumoral lymphoid stroma in the MNT and MNCA cases. In both tumors, PD‐1 (+) T cells were observed predominantly in the germinal centers, but not in the tumor nests, and were scattered in the peri‐tumoral lymphoid stroma (Fig. 2e,f). The cell density of PD‐1 (+) T cells in the peri‐tumoral lymphoid stroma was not significantly different between the MNT and MNCA cases (*P* = 0.295) (Fig. 3g; Table 2).

### PD‐L1 expression in tumor cells

**Figure 4:**
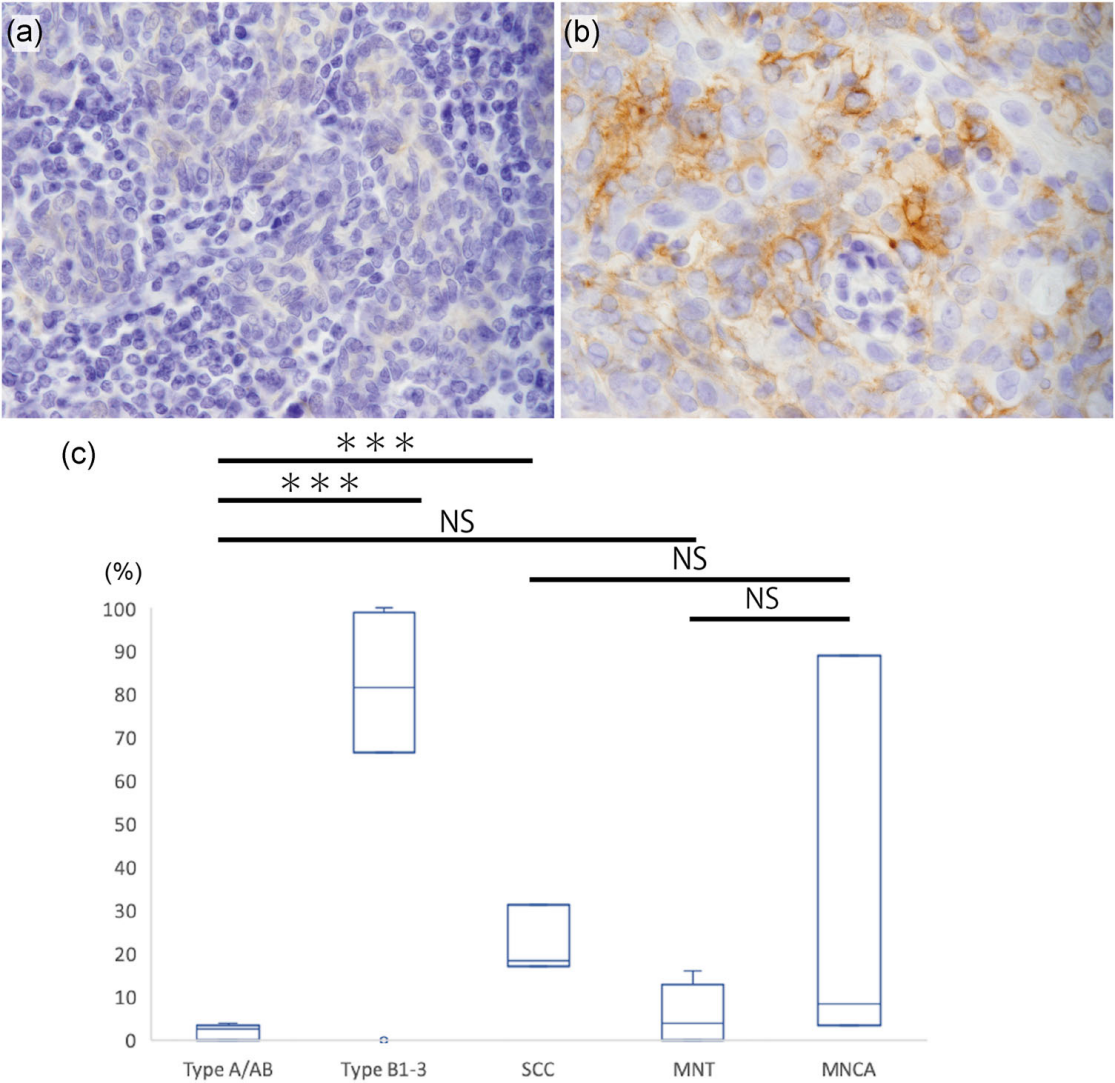
Expression of programmed death ligand 1 (PD‐L1) in tumor cells in micronodular thymoma with lymphoid stroma (MNT), micronodular thymic carcinoma with lymphoid stroma (MNCA), typical thymoma, and thymic squamous cell carcinoma (SCC). (**a,b**) Representative examples of immunohistochemical staining for PD‐L1. (**a**) MNT, (**b**) MNCA. (**c**) Positivity rate of PD‐L1 expression in tumor cells in type A and AB thymoma (*n* = 10), type B1‐3 thymoma (*n* = 11), thymic SCC (*n* = 3), MNT (*n* = 8) and MNCA (*n* = 3). Boxes represent the interquartile range, with the upper whisker indicating the 75th percentile and the lower whisker indicating the 25th percentile. The median values are indicated by a horizontal line. NS, not significant, ***P < 0.001.

We compared the tumor cell PD‐L1 expression in thymic epithelial neoplasms including MNT, MNCA, typical thymoma and thymic SCC (Fig. 4). The PD‐L1 expression levels were significantly higher in type B1‐3 thymoma and thymic SCC than in type A/AB thymoma, while no significant differences were observed between type A/AB thymoma and MNT, and between thymic SCC and MNCA (Fig. 4c). The PD‐L1 positivity rate tended to be higher in the MNCA cases (33.6%, range: 3.3–89.1%) than in the MNT cases (5.6%, range: 0–16.1%), but the difference was not significant (*P* = 0.112) (Fig. 4c; Supplementary Table S1). The PD‐L1 positivity rate in MNCA varied widely between cases.

## DISCUSSION

Both MNT and MNCA are characterized by the presence of abundant lymphoid stroma, suggesting the presence of a specific tumor immune microenvironment.^7^ While cytomorphologic and immunohistochemical features of tumor cells are different in MNT and MNCA, the histomorphologic features of lymphoid stroma in both tumors are similar. Because the characteristics of the immune microenvironment in MNT and MNCA have not been studied, we investigated the distribution and cell density of infiltrating CD8 (+) T cells, FOXP3 (+) T cells and PD‐1 (+) T cells and the PD‐L1 positivity rate in the tumor cells.

The clinical characteristics of cases in our cohort were compared with those noted in a previous meta‐analysis (Table 3).^4^ Neither metastasis nor tumor death has been reported in MNT patients in the previous studies and in our study.^2^, ^4^ In contrast, one tumor‐related death was reported among eight MNCA patients in the previous studies,^4^ whereas no case exhibited metastasis or tumor‐related death in our cohort. One possible reason for this difference could be the tumor stage. Most cases in our study were diagnosed at an early stage compared with cases in the previous studies (Table 3).^4^, ^5^ Although MNCA was suggested to be a malignant counterpart of MNT, it remains inconclusive whether MNCA is associated with a worse prognosis than MNT because of the scarcity of MNCA cases.^4^, ^5^

**Table 3:** Clinical summary of the findings of a previous meta‐analysis and the eight MNT and three MNCA cases in the present study

	Mneimneh *et al*.^3^		Present study	
	MNT (*n = 55*)	MNCA (*n = 8*)	MNT (*n = 8*)	MNCA (*n = 3*)
Age, mean and range (years)	64 (41–75)	60 (42–78)	63.3 (47–76)	64.0 (45–76)
Male‐to‐female ratio	1.4:1	5:3	4:4	2:1
Myasthenia gravis	3	0	0	0
Size, mean and range (mm)	60 (12–100)	55 (32–100)	39 (18–60)	18 (16–20)
No. of patients with follow‐up	27	6	8	3
Follow‐up period (months)	47 (6 days–180)	23 (3–39)	61.6 (14–120)	51.0 (13–92)
Patients who died of disease	0	1	0	0
Patients who died of other causes disease	5	0	0	0
Masaoka stage				
Stage I	31	3	2	2
Stage II	18	0	6	1
Stage III	1	3	0	0
Stage IV	0	1	0	0

MNCA, micronodular thymic carcinoma with lymphoid stroma; MNT, micronodular thymoma with lymphoid stroma.

In this study, the cell density of CD8 (+) T cells was higher in MNT, while the cell density of FOXP3 (+) T cells was higher in MNCA and consequently, the CD8/FOXP3 ratio was remarkably higher in MNT than in MNCA (Fig. 3). This tendency was observed in both the tumor nest and peri‐tumoral lymphoid stroma. CD8 (+) T cells are the major effector cells responsible for inducing apoptosis in the tumor immune microenvironment.^9^, ^17^ In various malignant tumors, including esophageal carcinoma and colorectal carcinoma, a high degree of CD8 (+) T cell infiltration was shown to correlate with better prognoses.^18^, ^19^, ^20^, ^21^, ^22^, ^23^, ^24^, ^25^, ^26^, ^27^ FOXP3 is a biomarker of regulatory T cells, which suppress the function of CD8 (+) cytotoxic T cells and reduce the level of antitumor immune activity.^11^, ^28^, ^29^, ^30^ As a result, a higher degree of FOXP3 (+) T cell infiltration correlates with a worse prognosis in various malignant tumors including hepatocellular carcinoma and breast carcinoma.^22^, ^23^, ^31^, ^32^, ^33^ These results suggest that antitumor immunoactivity is higher in MNT than in MNCA. In typical thymomas and thymic SCC, type B thymoma and thymic SCC presented a larger number of FOXP3 (+) T cells than did type A or AB thymoma,^34^ and the prognosis of type B thymoma and thymic SCC was worse than that of type A or AB thymoma in previous studies.^1^ In our study, the cell density of FOXP3 (+) T cells was significantly higher in MNCA than in MNT. This tendency may support the hypothesis that MNCA is the malignant counterpart of MNT. However, this hypothesis is largely based on the cytomorphologic and immunohistochemical features of MNCA, not on prognostic data from a large cohort. Our results suggested that MNCA may have a specific immune microenvironment that evades immunity, despite similar histomorphological features of the lymphoid stroma.

PD‐L1 binds to PD‐1 on CD8 (+) T cells and suppresses tumor immune response.^35^ Several studies focusing on PD‐L1 expression in tumor cells of thymic epithelial neoplasms have shown that the PD‐L1‐positivity rate in the tumor cells tends to be higher in type B thymoma and thymic SCC cases than in type A or AB thymoma cases.^34^, ^36^, ^37^, ^38^, ^39^, ^40^, ^41^, ^42^, ^43^ The results of PD‐L1 expression in typical thymoma and thymic SCC cases of the present study are similar to those obtained in previous studies. In this study, the PD‐L1 positivity rate in tumor cells and the cell density of PD‐1 (+) T cells in the peri‐tumoral lymphoid stroma did not differ significantly between the MNT and MNCA cases. These inconclusive results may be attributed to the small size of MNCA cases and the wide variation in the number of PD‐1 (+) T cells and PD‐L1 (+) tumor cells (Fig. 4c; Supplementary Table S1). PD‐1 is expressed not only on regulatory T cells but also on exhausted cytotoxic T cells, B cells, monocytes, natural killer T cells and dendritic cells.^44^ The PD‐1 expression in cell populations other than regulatory T cells may be another reason for the different distribution of FOXP3 (+) cells and PD‐1 (+) cells. Consequently, these results did not show a significant difference in the level of PD‐1/PD‐L1‐related immune tolerance between the two diseases.

The relationship between PD‐L1 expression and prognosis of thymoma and thymic carcinoma remains unclear, and the use of immune checkpoint inhibitors (ICIs) for treating thymoma or thymic SCC has not yet been established.^34^, ^36^, ^37^, ^38^, ^39^, ^41^, ^42^, ^43^, ^45^ In a review of ICI therapy for thymoma and thymic SCC patients, a high expression of PD‐L1 was associated with more effective response, and the incidence of side effects was higher in thymoma.^46^ In our study, a high expression of PD‐L1 was observed in one case of MNCA. Although no tumor recurrence or tumor‐related death was recorded for this patient, ICI therapy for patients with recurrent or unresectable MNCA with high PD‐L1 expression requires further investigation.

In conclusion, this study revealed that the cell density of CD8 (+) T cells in MNT was significantly higher than that in MNCA, while the cell density of FOXP3 (+) T cells in MNCA was significantly higher than that in MNT. These results suggest that antitumor immunoreactivity is lower in MNCA than in MNT.

## DISCLOSURE STATEMENT

None declared.

## AUTHOR CONTRIBUTIONS

HY and TI: Conception and design of the study. HY, MN, ST, YS, MI, HO and HT: Histological diagnosis. HY, YO, HO and MI: Data analysis. HY, YA, YE, SM, HK, IK and TI: Immunohistochemical analysis. All authors have read and approved the final version of the manuscript.

## Supporting information

**Figure S1** (**a**) Representative figure of immunohistochemistry using anti‐CD8 (brown) and anti‐AE1/AE3 antibody (blue) (MNT). (**b**) Red area indicates the peri‐tumoral lymphoid stroma. (**c**) Green area indicates a tumor nest. MNT, micronodular thymoma with lymphoid stroma; MNCA, micronodular thymic carcinoma with lymphoid stroma.

**Figure S2** Representative examples of immunohistochemical staining for CD8, FOXP3 and PD‐1 in the normal thymic tissue adjacent to tumors. (**a**) CD8 (+) T cells are observed in both the cortex (C) and medulla (M). (**b**) FOXP3 (+) T cells and (**c**) PD‐1, programmed death protein 1 (PD‐1) (+) T cells are mainly observed in the medulla, with small amounts in the cortex.

**Table S1** Positivity rate of PD‐L1 expression in 8 MNT and 3 MNCA.

Supplementary Information
