# Supplementary material for: Difference in the distribution of tumor‐infiltrating CD8+ T cells and FOXP3+ T cells between micronodular thymoma with lymphoid stroma and micronodular thymic carcinoma with lymphoid stroma
Source: Pathol Int. 2021 Apr 5;71(7):453–62. doi: 10.1111/pin.13099 (PMC8359975; doi:10.1111/pin.13099)
Supplement: Supplementary file 3 — Table S1 Positivity rate of PD‐L1 expression in 8 MNT and 3 MNCA. [file PIN-71-453-s001.docx]

**Supplementary Table 1**

**Positivity rate of PD-L1 expression in 8 MNT and 3 MNCA**

| Case | Diagnosis | PD-L1 positive rate (%) |
| --- | --- | --- |
| 1 | MNT | 0 |
| 2 | MNT | 0 |
| 3 | MNT | 15.5 |
| 4 | MNT | 0 |
| 5 | MNT | 5.4 |
| 6 | MNT | 5.1 |
| 7 | MNT | 16.1 |
| 8 | MNT | 2.8 |
| 9 | MNCA | 89.1 |
| 10 | MNCA | 8.3 |
| 11 | MNCA | 3.3 |

PD-L1, programmed death ligand 1; MNT, micronodular thymoma with lymphoid stroma; MNCA, micronodular thymic carcinoma with lymphoid stroma
