# Supplementary material for: Difference in the distribution of tumor‐infiltrating CD8+ T cells and FOXP3+ T cells between micronodular thymoma with lymphoid stroma and micronodular thymic carcinoma with lymphoid stroma
Source: Pathol Int. 2021 Apr 5;71(7):453–62. doi: 10.1111/pin.13099 (PMC8359975; doi:10.1111/pin.13099)
Supplement: Supplementary file 4 — Supplementary Information [file PIN-71-453-s004.docx]

**Supplementary Figure 1.**

(a) Representative figure of immunohistochemistry using anti-CD8 (brown) and anti-AE1/AE3 antibody (blue) (MNT). (b) Red area indicates the peri-tumoral lymphoid stroma. (c) Green area indicates a tumor nest. MNT, micronodular thymoma with lymphoid stroma; MNCA, micronodular thymic carcinoma with lymphoid stroma**.**

**Supplementary Figure 2.**

Representative examples of immunohistochemical staining for CD8, FOXP3, and PD-1 in the normal thymic tissue adjacent to tumors. (a) CD8 (+) T cells are observed in both the cortex (C) and medulla (M). (b) FOXP3 (+) T cells and (c) PD-1 (+) T cells are mainly observed in the medulla, with small amounts in the cortex. PD-1, programmed death protein 1.
